# Supplementary material for: New Auranofin Analogs with Antibacterial Properties against Burkholderia Clinical Isolates
Source: Antibiotics (Basel). 2021 Nov 24;10(12):1443. doi: 10.3390/antibiotics10121443 (PMC8697972; doi:10.3390/antibiotics10121443)
Supplement: Supplementary file 1 [file antibiotics-10-01443-s001.zip › antibiotics-1444264-supplementary/MS-40_MS-40S Supplemental_v2.pdf]

Table S1. Minimum inhibitory concentrations (MICs) of group one auranofin derivatives against a panel of *Burkholderia cepacia* complex (Bcc) bacteria.

Organisms	MIC (μg/mL)				
	Auranofin	WB-18-FI3683	WB-16-EO9899	WB-19-HG5899	WB-19-HB2664
B. cenocepacia K56-2	>128	>128	>128	>128	>128
B. lata BCC6	128	128	128	128	128
B. contaminans MF16	128	128	128	128	128
B. contaminans FFH-2050MA	128	128	128	64	64
B. dolosa CEP021	>128	>128	>128	>128	>128
B. multivorans ATCC 17616	128	128	128	128	128
B. cenocepacia 140485	128	64	32	128	128
B. ubonensis LMG 20358	>128	>128	>128	128	128
B. contaminans FFH-4004	>128	>128	>128	128	128
B. mallei China 5 (NBL 4)	0.25	ND	ND	ND	ND
B. mallei Ivan (NCTC 10230)	0.5	ND	ND	ND	ND
B. mallei China 7 (NBL 7)	1	ND	ND	ND	ND
B. pseudomallei 1710b	64	ND	ND	ND	ND
B. pseudomallei MSHR465a	64	ND	ND	ND	ND
B. pseudomallei HBPUB10134a	64	ND	ND	ND	ND

The reported MIC values are from three biological replicates. In the case a 2-fold difference occurred, the higher value is reported.

Table S2. Bacterial species and strains used in this study.

Organism	Features	Source
B. cenocepacia K56-2	Cystic fibrosis clinical isolate, ETI2 lineage	(1)
B. lata BCC6	Isolated from mirror defogger	C. Deschênes
B. contaminans MF16	Clinical Isolate	(2)
B. contaminans FFH-2050MA	Clinical Isolate	Hospital de Niños Ricardo Gutierrez, Buenos Aires, Argentina
B. dolosa CEP021	Clinical isolate	D. Speert
B. multivorans ATCC 17616	Environmental Isolate	Dr. Tiedge
B. cenocepacia 140485	Clinical Isolate	National Microbiology Laboratory
B. ubonensis LMG 20358	Surface soil isolate	(3, 4)
B. contaminans FFH-4004	Clinical isolate	CF Isolate from Argentina
B. mallei China 5 (NBL 4)	Animal Isolate	BEI Resources
B. mallei Ivan (NCTC 10230)	Animal Isolate	BEI Resources
B. mallei China 7 (NBL 7)	Clinical Isolate	BEI Resources
B. pseudomallei 1710b	Clinical Isolate	BEI Resources
B. pseudomallei MSHR465a	Clinical Isolate	BEI Resources
B. pseudomallei HBPUB10134a	Clinical Isolate	BEI Resources
B. pseudomallei MSHR305	Clinical Isolate	BEI Resources
Stenotrophomonas maltophilia DH57	Clinical isolate	ATCC
Stenotrophomonas maltophilia K279a	Clinical isolate	ATCC
Pseudomonas. aeruginosa PA01	Reference strain	A. Kumar
Pseudomonas aeruginosa PA7	MDR, non-respiratory isolate	A. Kumar, (5)
Escherichia coli 120955	ESBL-producing clinical isolate	G. Zhanel

Escherichia vulneris CEP511	Clinical isolate	D. Speert
Klebsiella pneumoniae 120310	ESBL-producing clinical isolate	G. Zhanel
Acinetobacter baumannii ATCC 17978	Reference strain	A. Kumar
Staphylococcus aureus ATCC 27700	Reference Strain	K. Lovetri
Staphylococcus aureus 107094	MRSA, clinical isolate	Paediatric Hospital of Buenos Aires, Argentina

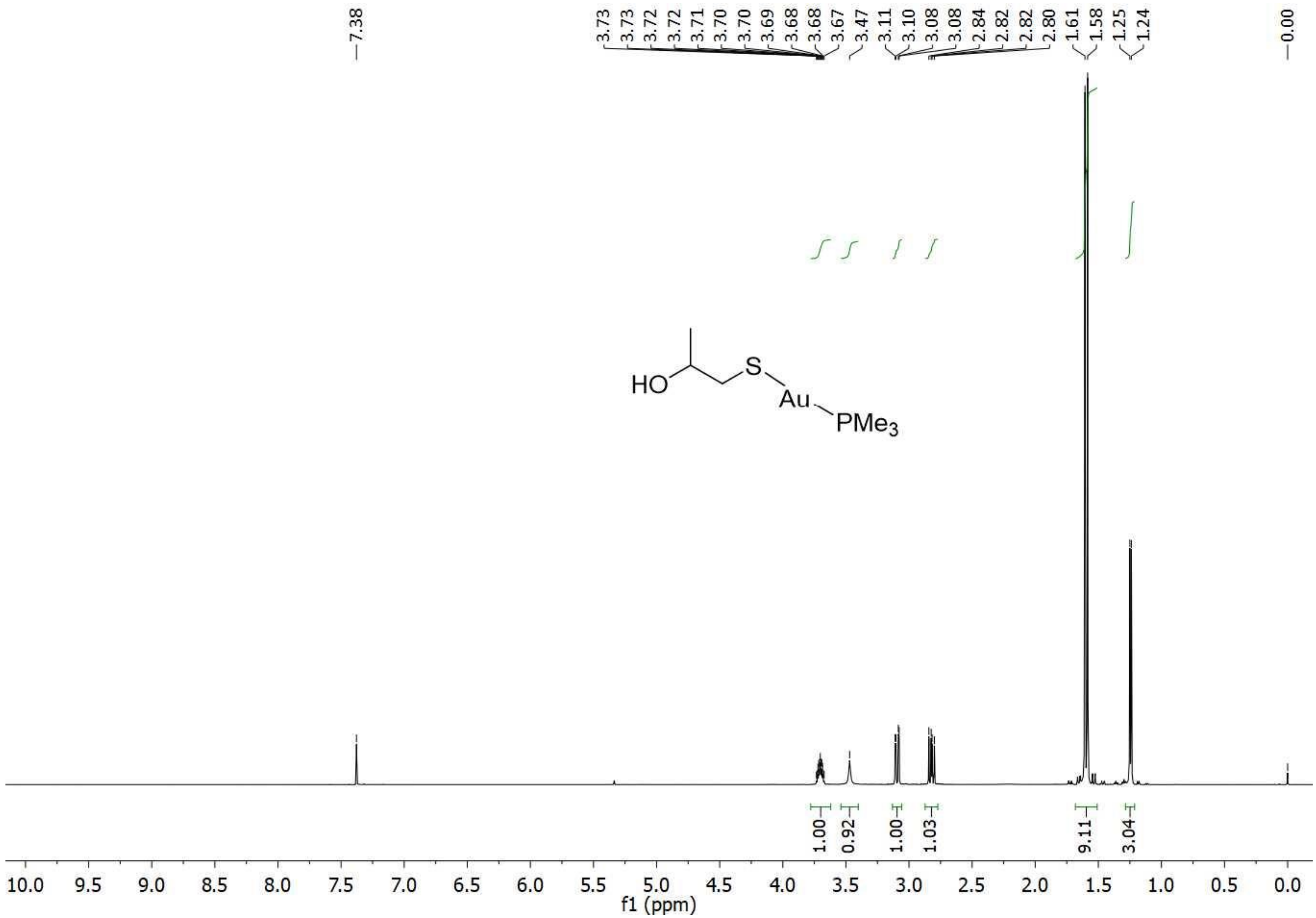

Figure S1. ^{1}H NMR spectrum of compound **WB-19-HL4170** (MS-40S) in $CDCl_3$.

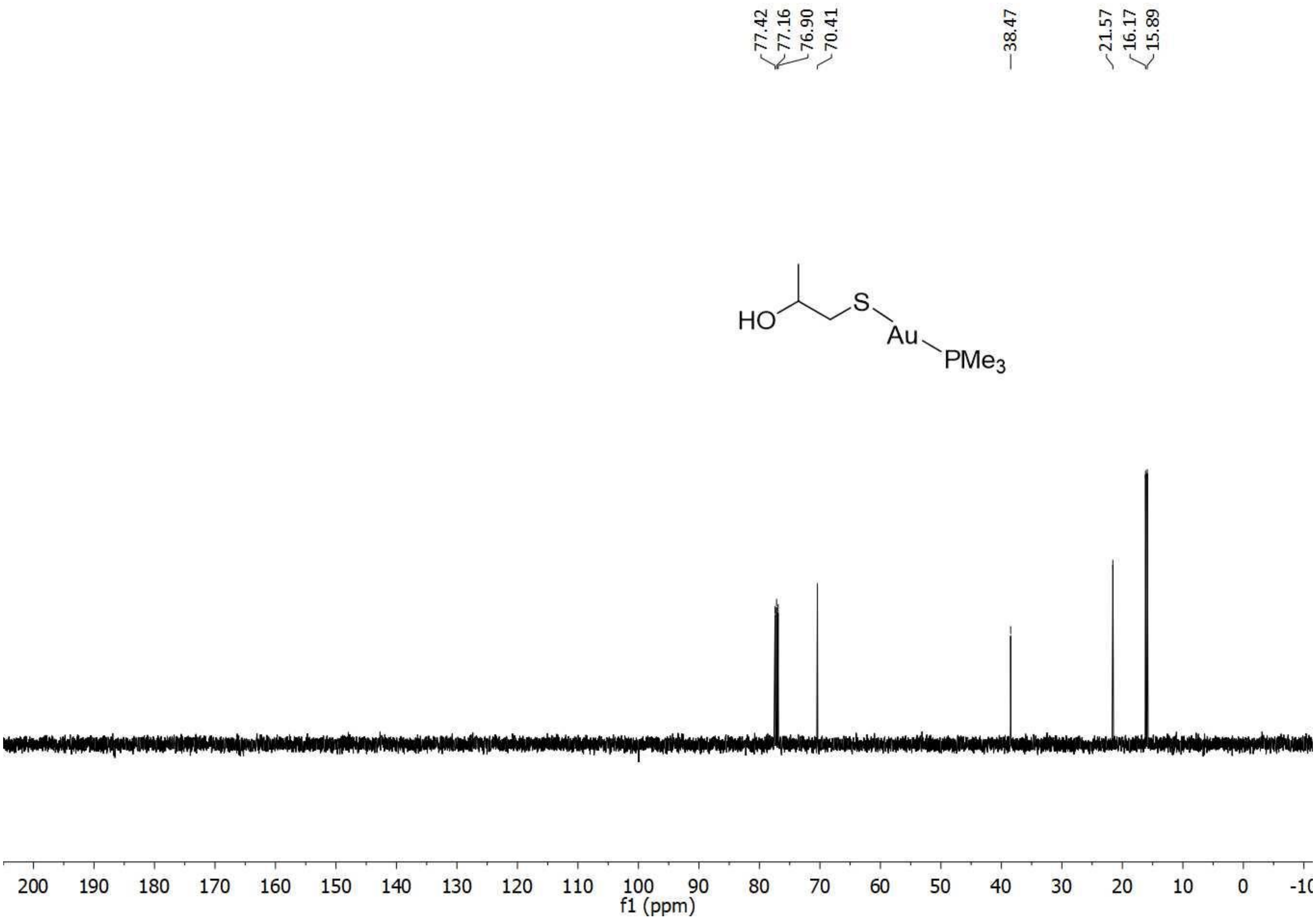

Figure S2. ^{13}C NMR spectrum of compound **WB-19-HL4170** (MS-40S) in $CDCl_3$.

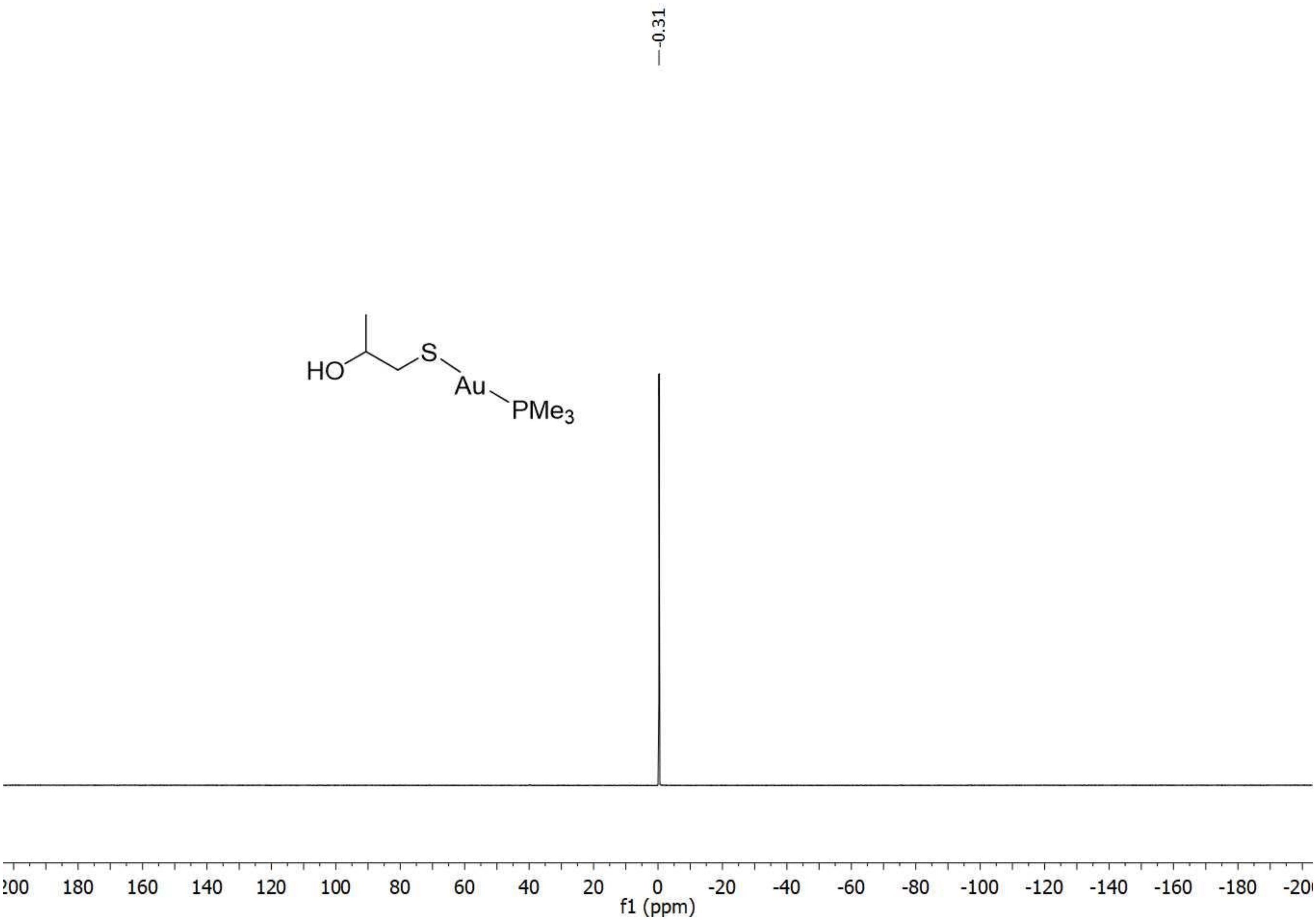

Figure S3. ^{31}P NMR spectrum of compound **WB-19-HL4170** (MS-40S) in $CDCl_3$.

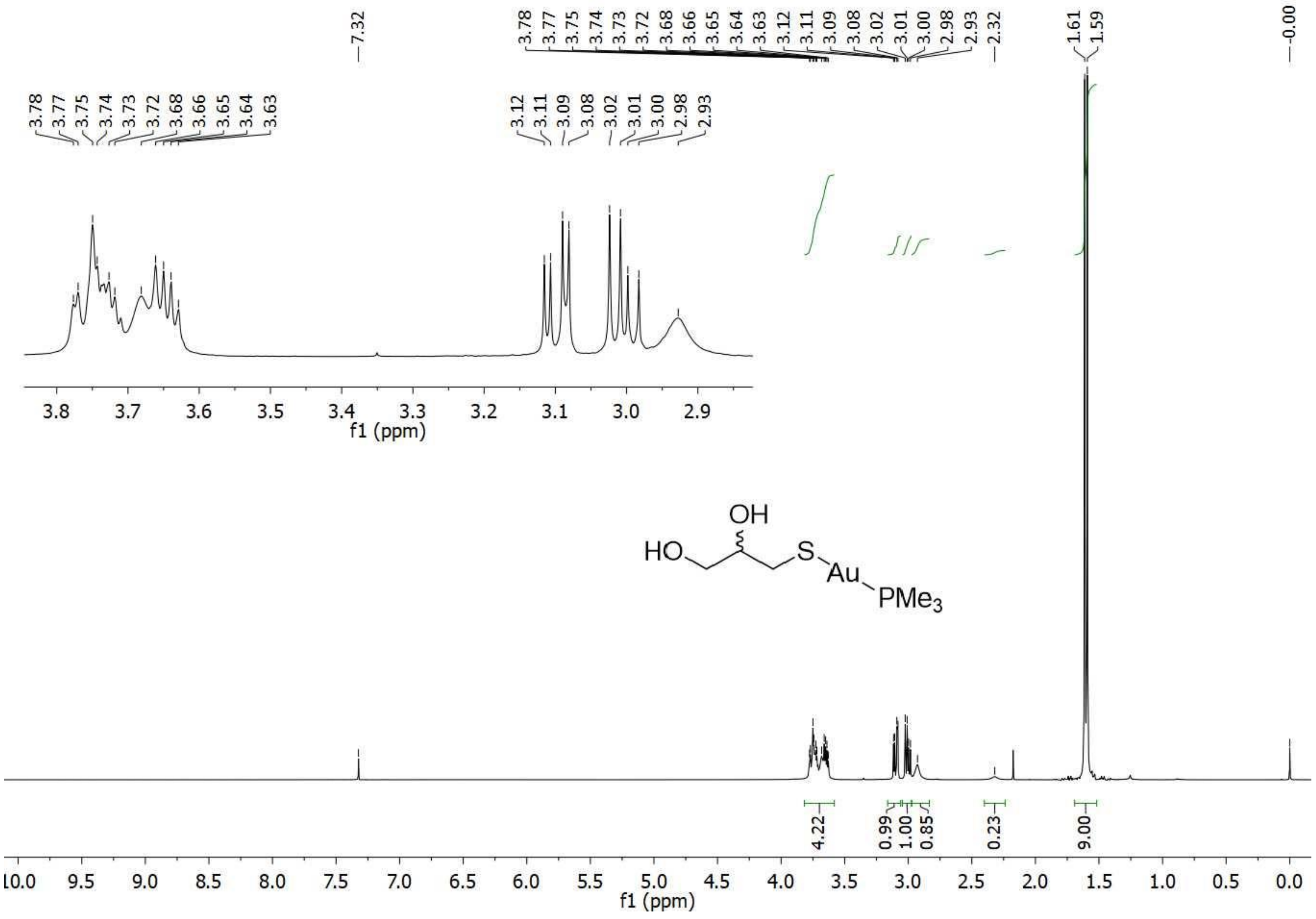

Figure S4. ^{1}H NMR spectrum of compound **WB-19-HL4171** in $CDCl_3$.

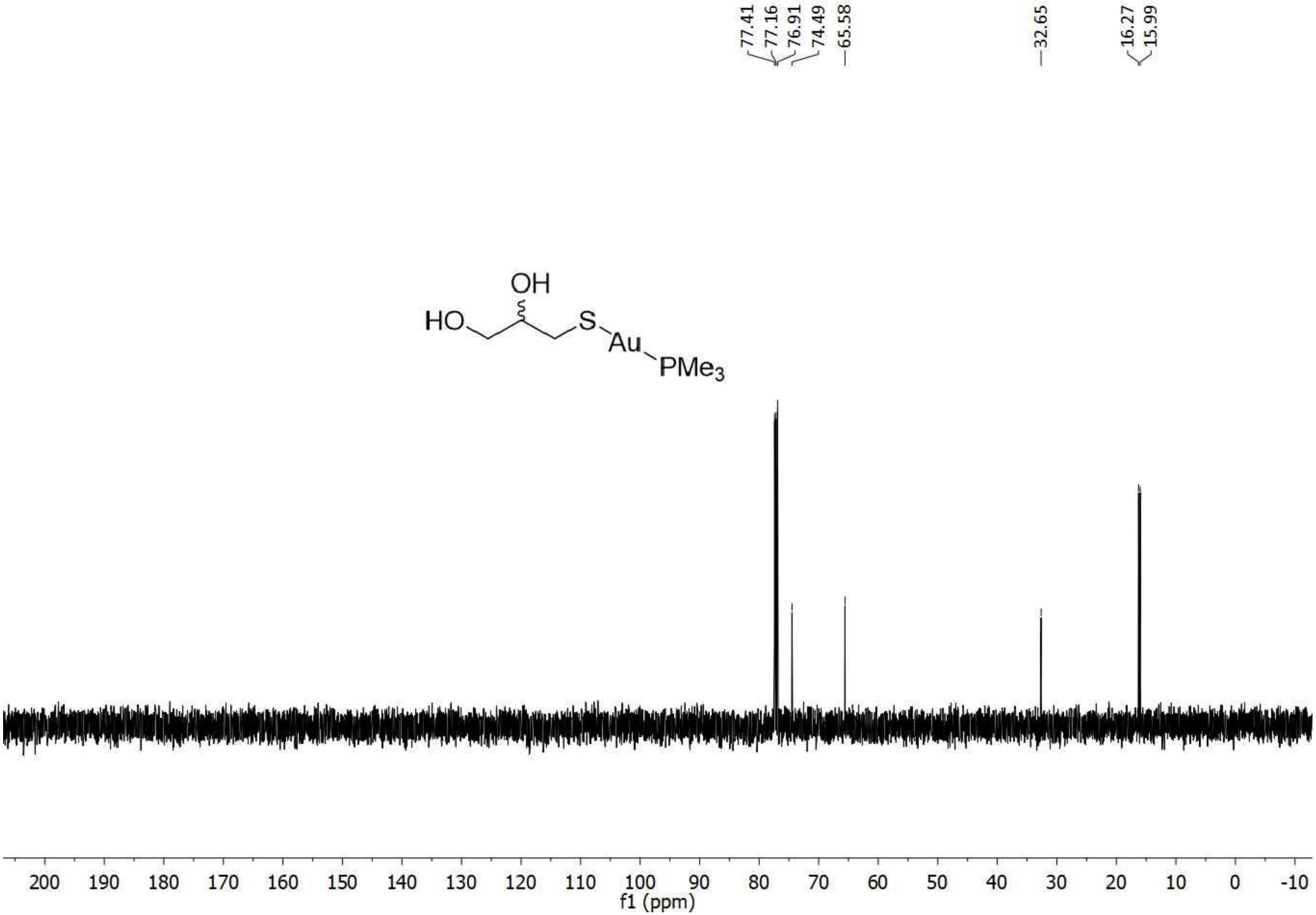

Figure S5. ^{13}C NMR spectrum of compound **WB-19-HL4171** in $CDCl_3$.

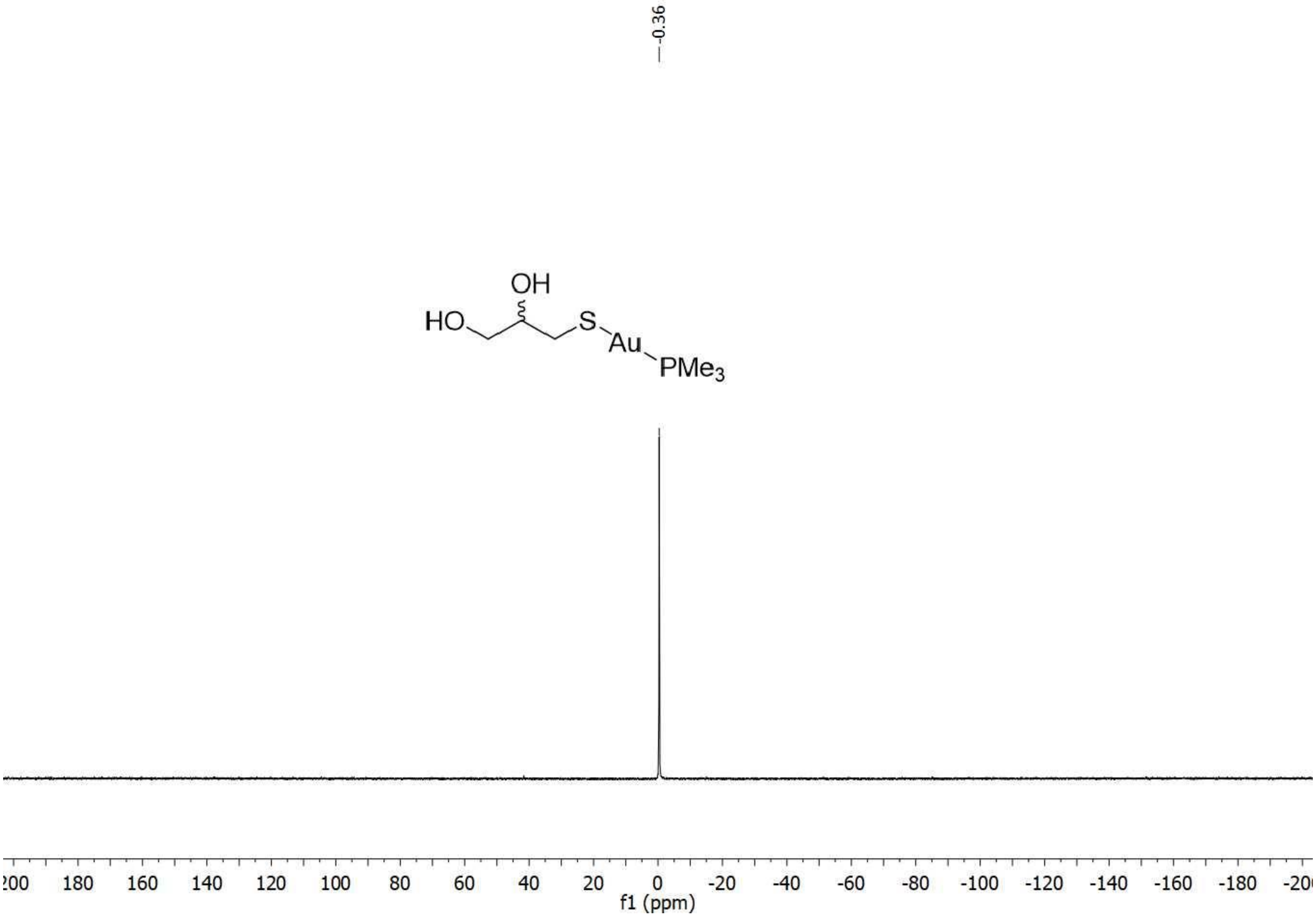

Figure S6. ^{31}P NMR spectrum of compound **WB-19-HL4171** in $CDCl_3$.

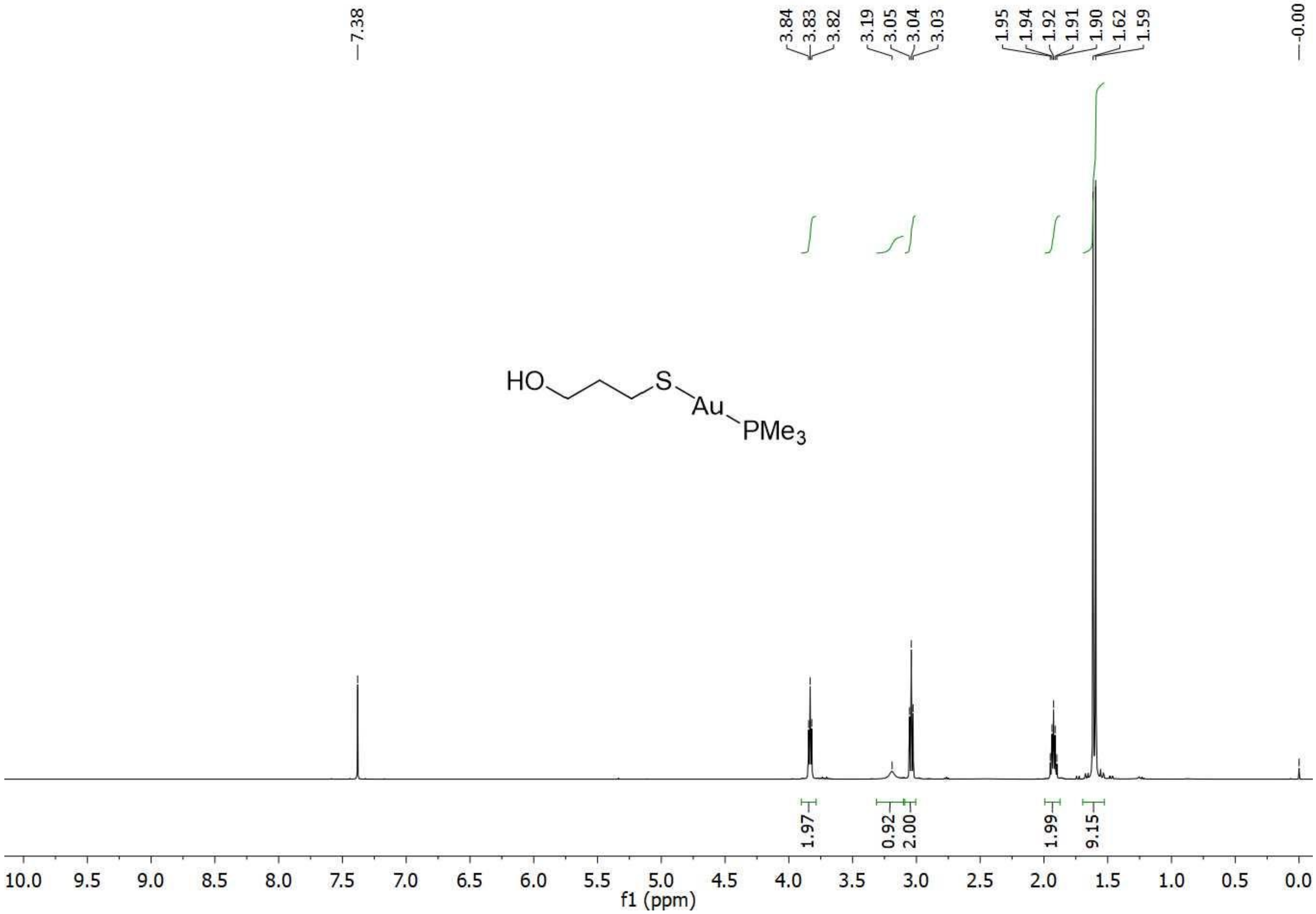

Figure S7. ^{1}H NMR spectrum of compound **WB-19-HL4172** in $CDCl_3$.

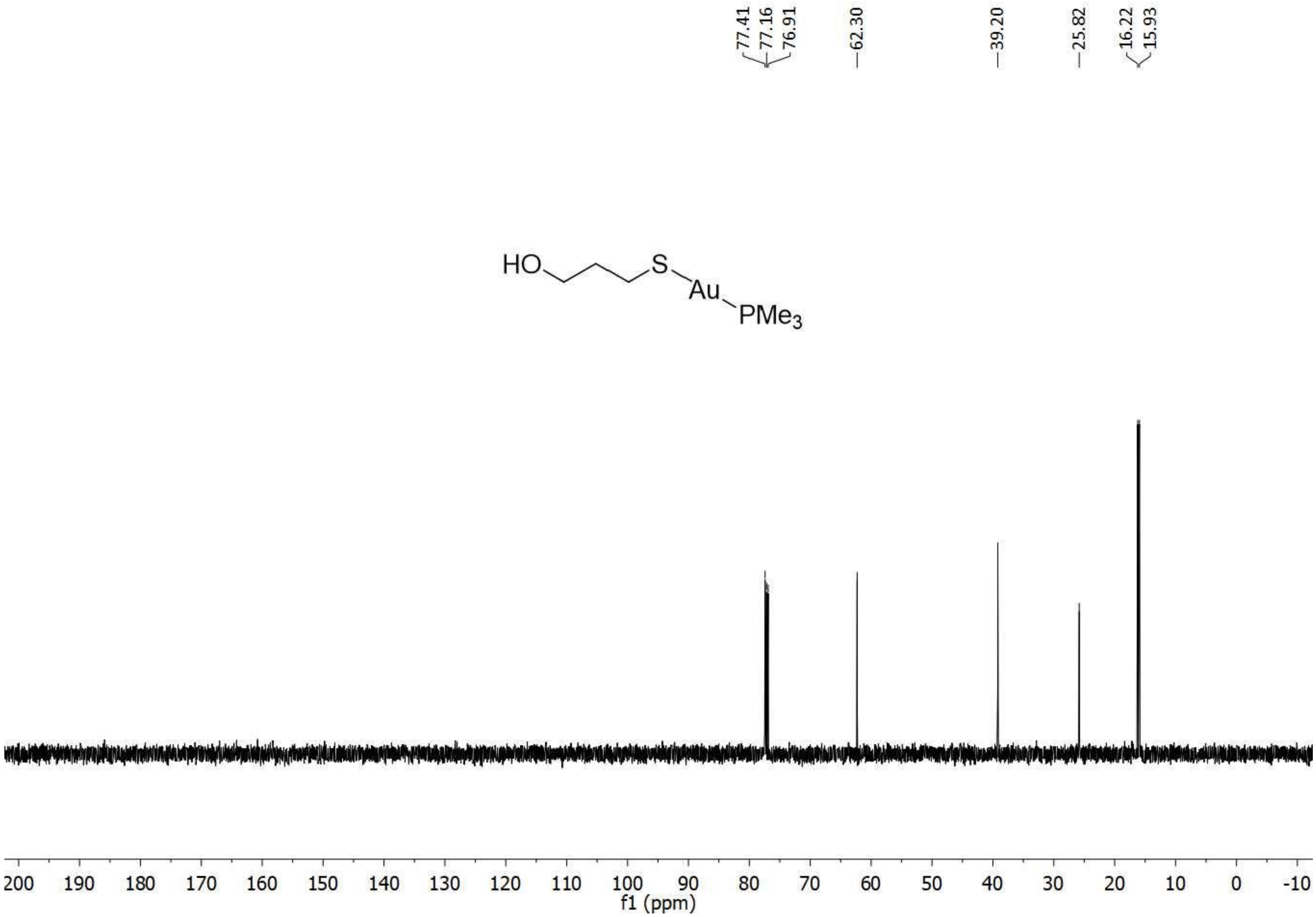

Figure S8. ^{13}C NMR spectrum of compound **WB-19-HL4172** in $CDCl_3$.

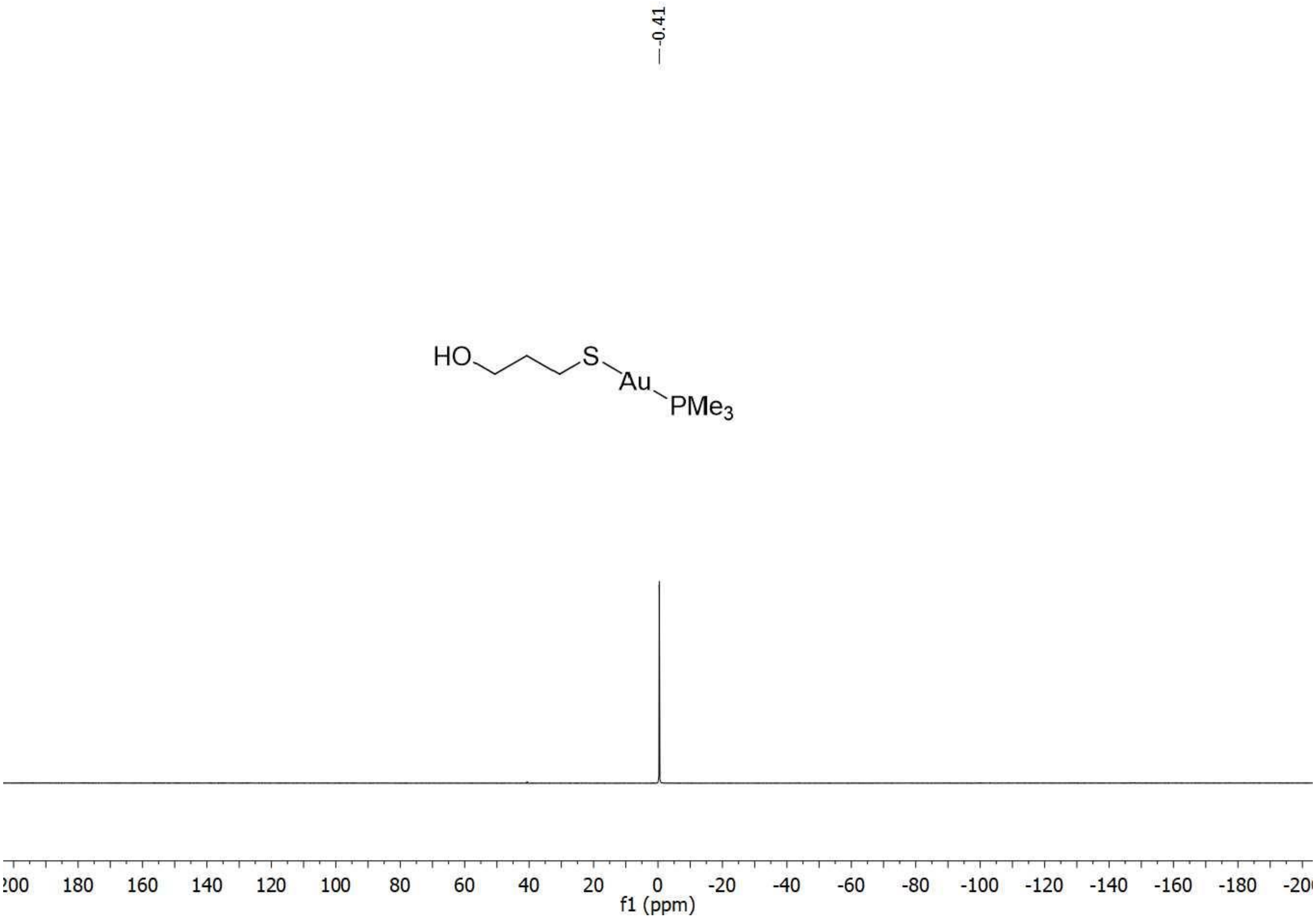

Figure S9. ^{31}P NMR spectrum of compound **WB-19-HL4172** in $CDCl_3$.

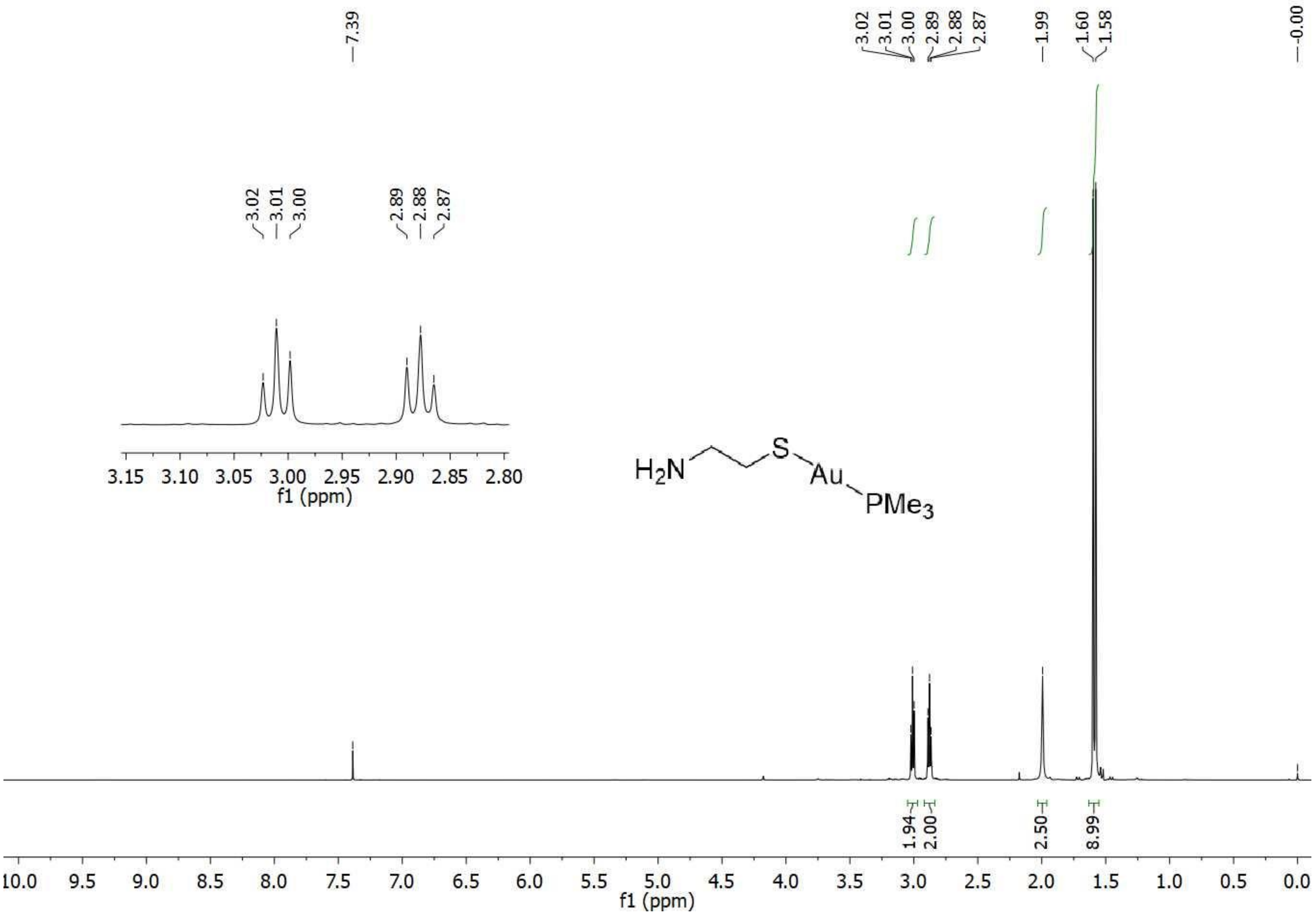

Figure S10. ^{1}H NMR spectrum of compound **WB-19-HL4181** in $CDCl_3$.

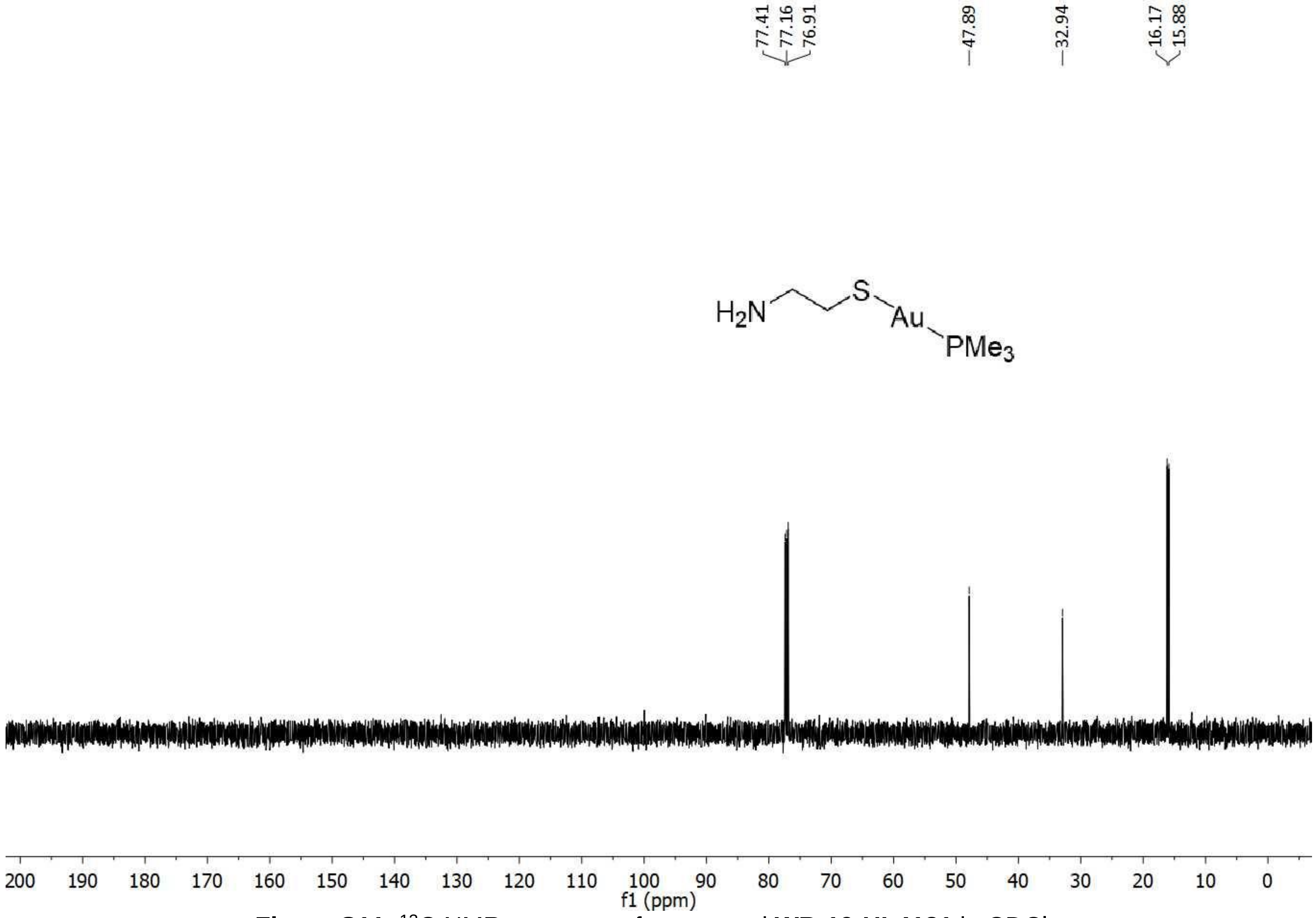

Figure S11. ^{13}C NMR spectrum of compound **WB-19-HL4181** in $CDCl_3$.

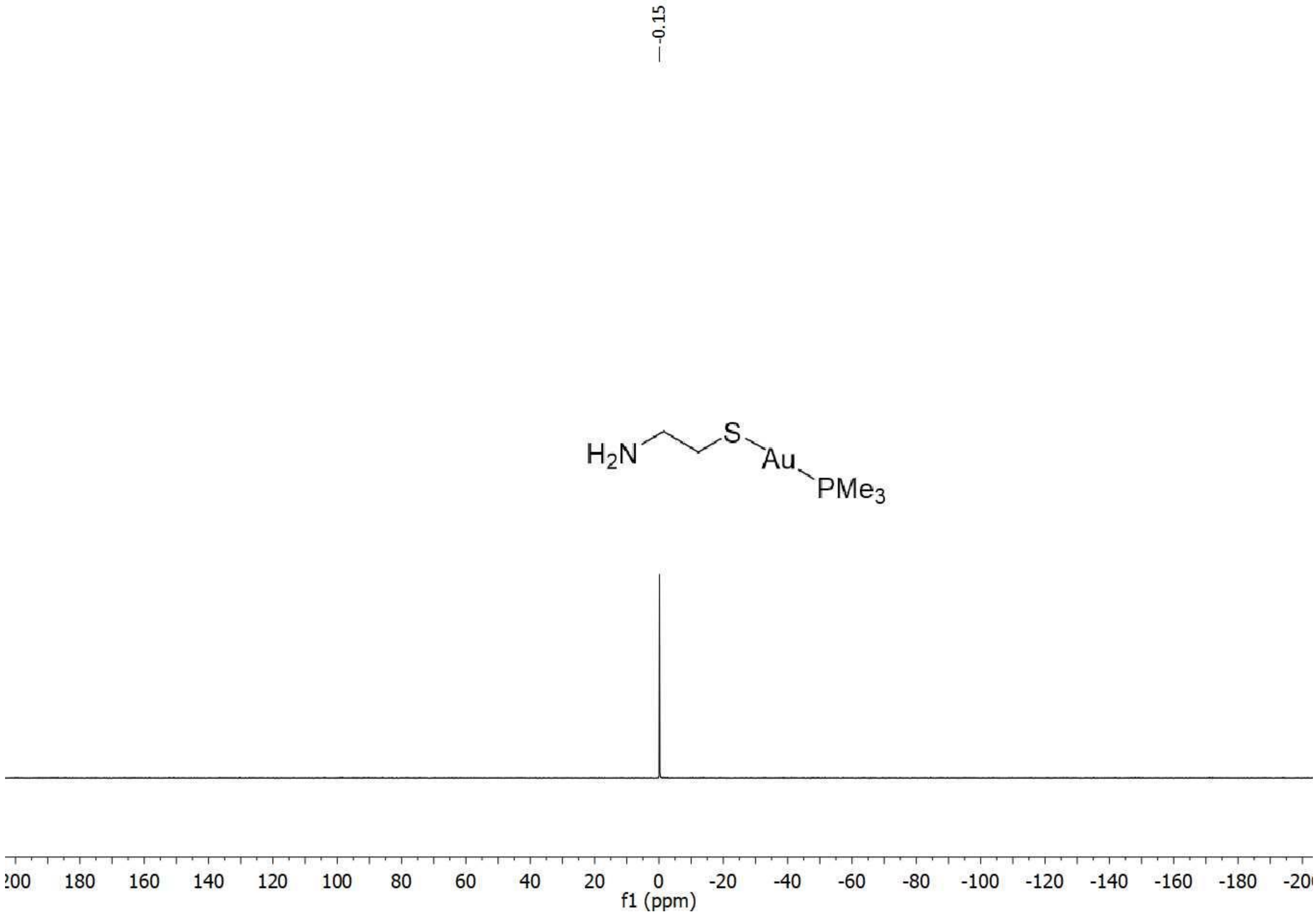

Figure S12. ^{31}P NMR spectrum of compound **WB-19-HL4181** in $CDCl_3$.

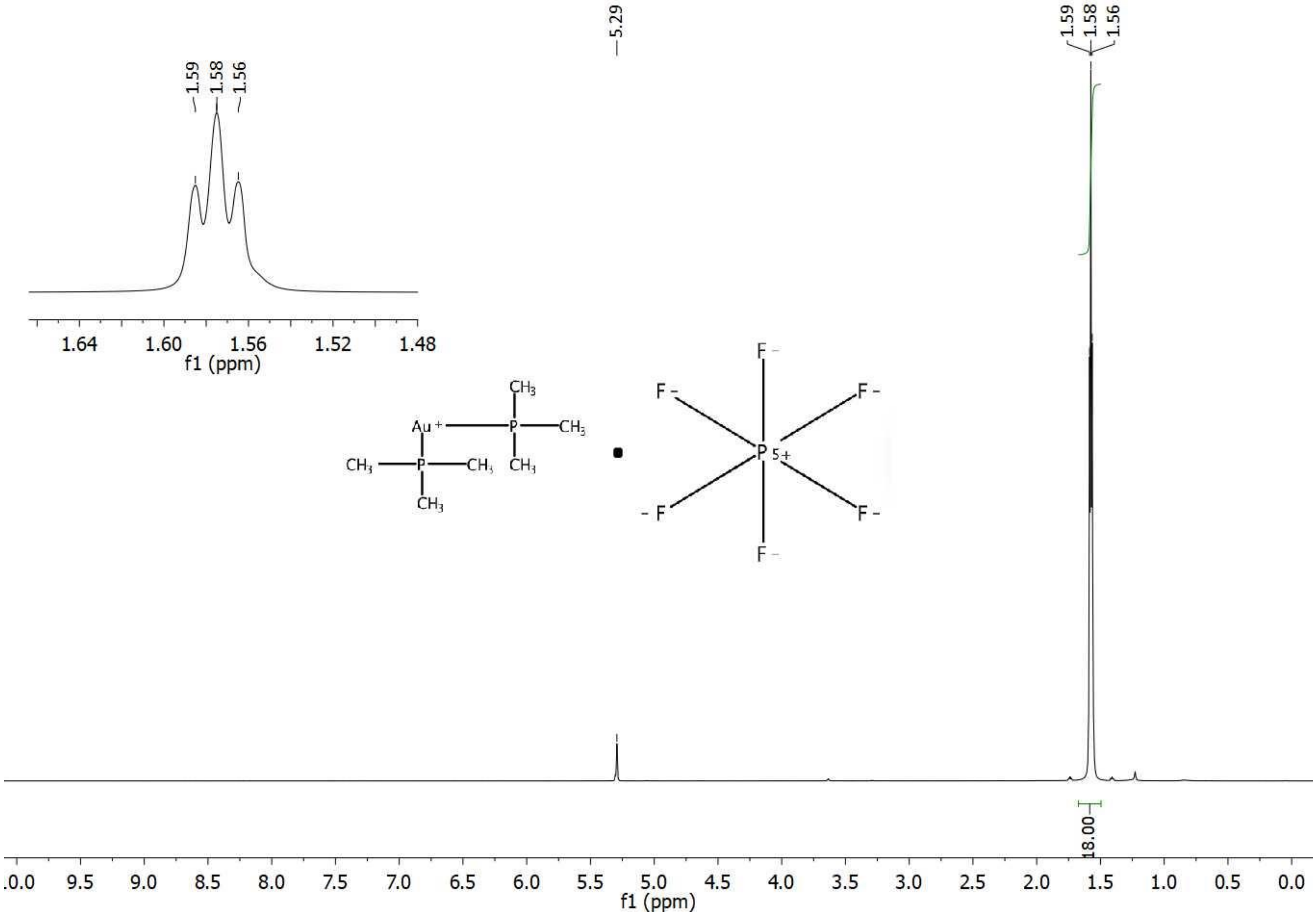

Figure S13. ^{1}H NMR spectrum of compound **WB-19-HL4121a** in CD_2Cl_2.

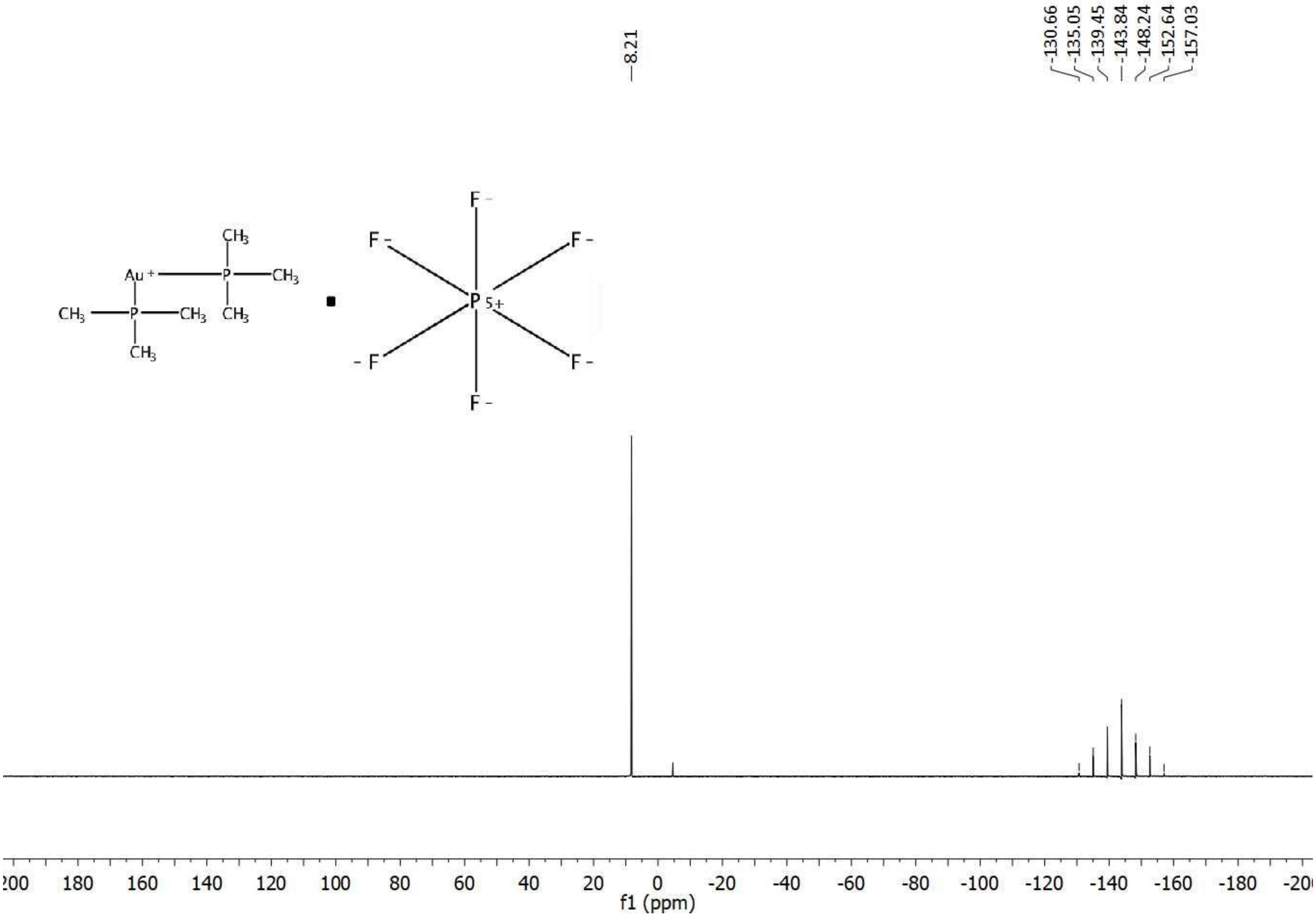

Figure S14. ^{31}P NMR spectrum of compound **WB-19-HL4121a** in CD_2Cl_2.

References

1. Darling P, Chan M, Cox AD, Sokol PA. 1998. Siderophore production by cystic fibrosis isolates of *Burkholderia cepacia*. Infection and immunity 66:874–877.

2. Nunvar J, Kalferstova L, Bloodworth RA, Kolar M, Degrossi J, Lubovich S, Cardona ST, Drevinek P. 2016. Understanding the Pathogenicity of *Burkholderia contaminans*, an Emerging Pathogen in Cystic Fibrosis. PloS one 11:e0160975.

3. Schmidt S, Blom JF, Pernthaler J, Berg G, Baldwin A, Mahenthiralingam E, Eberl L. 2009. Production of the antifungal compound pyrrolnitrin is quorum sensing-regulated in members of the *Burkholderia cepacia* complex. Environ Microbiol 11:1422–1437.

4. Yabuuchi E, Kawamura Y, Ezaki T, Ikedo M, Dejsirilert S, Fujiwara N, Naka T, Kobayashi K. 2000. *Burkholderia uboniae* Sp. Nov., L -Arabinose-Assimilating but Different from *Burkholderia thailandensis* and *Burkholderia vietnamiensis*. Microbiology and Immunology 44:307–317.

5. Roy PH, Tetu SG, Larouche A, Elbourne L, Tremblay S, Ren Q, Dodson R, Harkins D, Shay R, Watkins K, Mahamoud Y, Paulsen IT. 2010. Complete Genome Sequence of the Multiresistant Taxonomic Outlier Pseudomonas aeruginosa PA7. PLOS ONE 5:e8842.
